# Induction of Neuronal Differentiation of Rat Muscle-Derived Stem Cells *in Vitro* Using Basic Fibroblast Growth Factor and Ethosuximide

**DOI:** 10.3390/ijms14046614

**Published:** 2013-03-25

**Authors:** Mi Lan Kang, Jin Seon Kwon, Moon Suk Kim

**Affiliations:** Department of Molecular Science and Technology, Ajou University, Suwon 443-759, Korea; E-Mails: milan511@snu.ac.kr (M.L.K.); jinseon@ajou.ac.kr (J.S.K.)

**Keywords:** adult stem cells, muscle derived stem cells, bFGF, ethosuximide, neurogenesis, neurons

## Abstract

Several studies have demonstrated that basic fibroblast growth factor (bFGF) can induce neural differentiation of mesenchymal stem cells. In this study, we investigated the neural differentiation of muscle-derived stem cells (MDSCs) following treatment with bFGF and ethosuximide, a small molecule used as an anticonvulsant in humans. Stem cells isolated from rat skeletal muscle (rMDSCs) were pre-induced by culturing with 25 ng/mL bFGF for 24 h and then were transferred to a medium supplemented with or without 4 mM ethosuximide. Neuronal differentiation was assessed by immunocytochemical and western blotting analyses of marker expression. Immunocytochemistry of rMDSCs treated with bFGF and ethosuximide identified abundant cells expressing neuronal markers (TuJ1, neuron-specific class III β-tubulin; NeuN, neuronal nuclear antigen; and NF-MH; neurofilament M and H). Olig2 (oligodendrocyte transcription factor 2)-positive cells were also observed, indicating the presence of oligodendrocyte lineage cells. These findings were substantiated by western blotting analysis of marker proteins. In particular, the expression of NeuN and TuJ1 was significantly higher in rMDSCs treated with ethosuximide and bFGF than in cells stimulated with bFGF alone (NeuN, *p* < 0.05 and TuJ1, *p* < 0.001). Expression of the astrocyte marker GFAP (glial fibrillary acidic protein) was not detected in this study. Collectively, the results showed that treatment with bFGF and ethosuximide induced effective transdifferentiation of rMDSCs into cells with a neural-like phenotype. Notably, rMDSCs treated with a combination of bFGF plus ethosuximide showed enhanced differentiation compared with cells treated with bFGF alone, implying that ethosuximide may stimulate neuronal differentiation.

## 1. Introduction

Adult stem cells have a much lower capacity than embryonic stem cells to self-renew and differentiate along multiple lineage pathways. However, adult stem cells are immunocompatible, and their use is not restricted by the ethical concerns associated with embryo-derived cells. Mesenchymal stem cells can be easily isolated from many adult tissues such as bone marrow, periosteum, trabecular bone, adipose tissue, synovium, skeletal muscle, and deciduous teeth [1,2], and are therefore commonly used to study stem cell multipotency. Skeletal muscle-derived stem cells (MDSCs) have long been considered to be unipotent stem cells capable of differentiating only into myogenic lineage cells [3]. However, recent evidence supports the existence of multipotent MDSCs that can participate in the healing and regeneration of several tissues [4–12]. These reports demonstrate that MDSCs can differentiate into various cell lineages using defined culture conditions, and that large quantities of differentiated cells could be obtained *in vitro*. Therefore, MDSCs could be an excellent source of differentiated cell types for tissue engineering and cell therapies.

Despite the remarkable achievements of medical research, the treatment of central nervous system diseases remains challenging. Neural stem cells (NSCs) are capable of self-renewal and differentiation into neurons, astrocytes, and oligodendrocytes. NSC transplantation has shown great potential for the therapy of neurological disorders; however, NSCs commonly cultured *in vitro* as neurospheres do not behave as stem cells when transplanted back into the brain [13,14]. NSCs proliferate slowly throughout life and only small numbers persist in the adult brain. Moreover, these cells are difficult to isolate from human brain biopsy or autopsy samples. Thus, the ease of isolation and differentiation potential of MDSCs makes them an attractive possible source of functional neuronal or glial cells that could be used in cell therapy of neurological disorders. Indeed, several studies have demonstrated that MDSCs can undergo neuroectodermal differentiation to neurons and astrocytes [5–12].

In this study, we investigated the neural differentiation potential of rat MDSCs (rMDSCs) treated with basic fibroblast growth factor (bFGF) and ethosuximide. Several studies have demonstrated that bFGF can induce neural differentiation of stem cells derived from distinct mesenchymal tissues, including endometrial [15] and adipose [16] tissues. Ethosuximide is a small heterocyclic ring compound of the succinimide class and is used clinically to treat absence seizures in humans [17]. The compound primarily acts by blocking T-type voltage-gated calcium channels in thalamic neurons [18]. To the best of our knowledge, this is the first evidence that ethosuximide induces neurogenesis of rMDSCs *in vitro*, suggesting that ethosuximide might be useful in inducing neural differentiation of human MDSCs.

## 2. Results and Discussion

**Figure 1:**
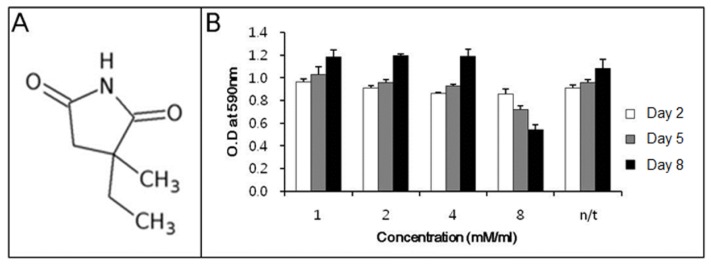
Structure of ethosuximide and cytotoxicity to rMDSCs. (**A**) The structure of ethosuximide, which is chemically designated as α-ethyl-α-methyl-succinimide (3-ethyl-3-methyl-pyrrolidine-2,5-dione; (**B**) Ethosuximide cytotoxicity was measured by the MTT assay. rMDSC neurogenesis was induced by treatment with 25 ng/mL bFGF for 24 h, and the medium was then replaced with medium containing the indicated concentrations of ethosuximide. MTT assays were performed after a total of 2, 5, or 8 days incubation.

Before determining the ability of ethosuximide as a neurogenic agent, we evaluated its potential cytotoxicity to rMDSCs. There have been no previous reports on the cytotoxic or genotoxic effects of ethosuximide on rMDSCs. Ethosuximide is a succinimide derivative (Figure 1A) and is highly soluble in aqueous solution. rMDSCs were incubated in the presence or absence of ethosuximide (1, 2, 4, and 8 mM), and the MTT assay was performed at 2, 5, or 8 days after the culture initiation of culture. As shown in Figure 1B, ethosuximide was toxic to rMDSCs at 8 mM but not at lower concentrations. Cells treated with 8 mM ethosuximide did not proliferate and underwent necrosis after 2 days. However, cells treated with lower concentrations of ethosuximide proliferated well and showed no evidence of cell death over the 8-day incubation period.

**Figure 2:**
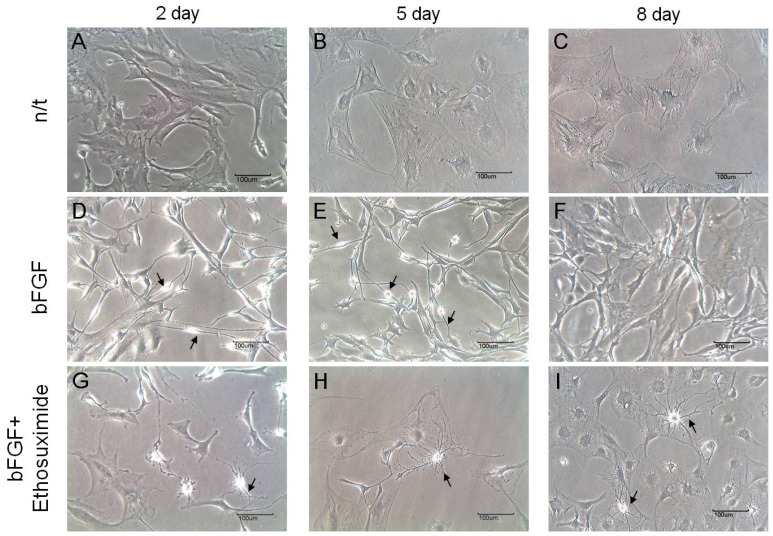
Inverted microscope photograph of morphology of rMDSCs. First column, 2 days after pre-induction of neurogenesis; second column, 5 days after pre-induction of neurogenesis; third column, 8 days after pre-induction of neurogenesis. First line, the rMDSCs without any stimulant; second line, the rMDSCs with bFGF only; third line, the rMDSCs with pre-induction of bFGF for 24 h and then ethosuximide 4 mM/mL. Magnification is ×200 and scale bar represents 100 μm.

The morphological changes observed in the cultures of rMDSCs treated with bFGF alone or with both bFGF and ethosuximide are shown in Figure 2. Cells stimulated with bFGF for 24 h and then transferred to medium appeared similar to γ-aminobutyric acid (GABA) neurons, which secrete GABA as their primary neurotransmitter (arrows in Figure 2D,E). On the other hand, the morphology of rMDSCs treated with both bFGF and ethosuximide was more reminiscent of oligodendrocytes (arrows in Figure 2G–I). Oligodendrocytes are a variety of neuroglia that play a supporting role for neurons.

**Figure 3:**
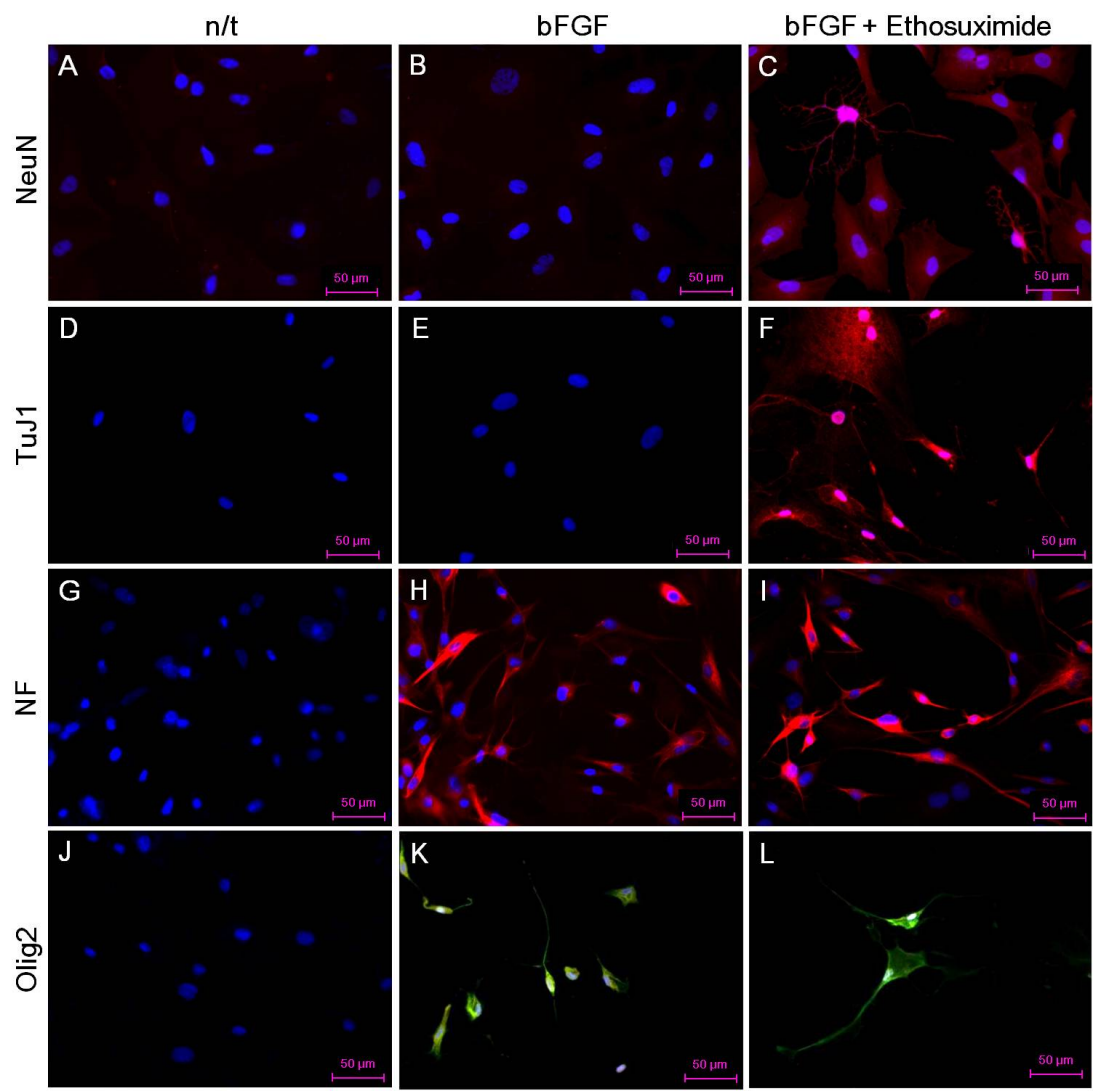
Fluorescence immunostaining of rMDSCs. First column, rMDSCs without any stimulant; second column, rMDSCs differentiated with bFGF; third column, rMDSCs differentiated with bFGF and ethosuximide. First line, NeuN fluorescence immunostaining; second line, TuJ1 fluorescence immunostaining; third line, NF fluorescence immunostaining; fourth line, Olig2 fluorescence immunostaining. Each immunostaining performed with rMDSCs at 5 days after pre-induction of neurogenesis. Magnification is ×400 and scale bar represents 50 μm.

The effect of ethosuximide treatment on lineage-specific differentiation of rMDSCs in culture was monitored by immunocytochemical detection of the neuronal markers NeuN and TuJ1, the oligodendrocyte marker Olig2, and the neurofilament marker NF-MH. These neural markers were not detected in rMDSCs cultured in the absence of bFGF and ethosuximide (Figure 3A,D,G,J). The rMDSCs stimulated with bFGF alone contained cells expressing NF and Olig2 but not NeuN or TuJ1 (Figure 3B,E,H,K). However, treatment with both bFGF and ethosuximide induced rMDSCs to differentiate into cells expressing NeuN, TuJ1, and NF (Figure 3C,F,I) and Olig2 (Figure 3L), indicating the presence of neuronal lineage and oligodendrocyte lineage cells, respectively. Notably, a larger proportion of cells in these cultures expressed NeuN and TuJ1 than Olig2. These results therefore suggest that rMDSCs treated with ethosuximide differentiated mainly into cells with neural-like characteristics, implying that ethosuximide may stimulate neural differentiation.

**Figure 4:**
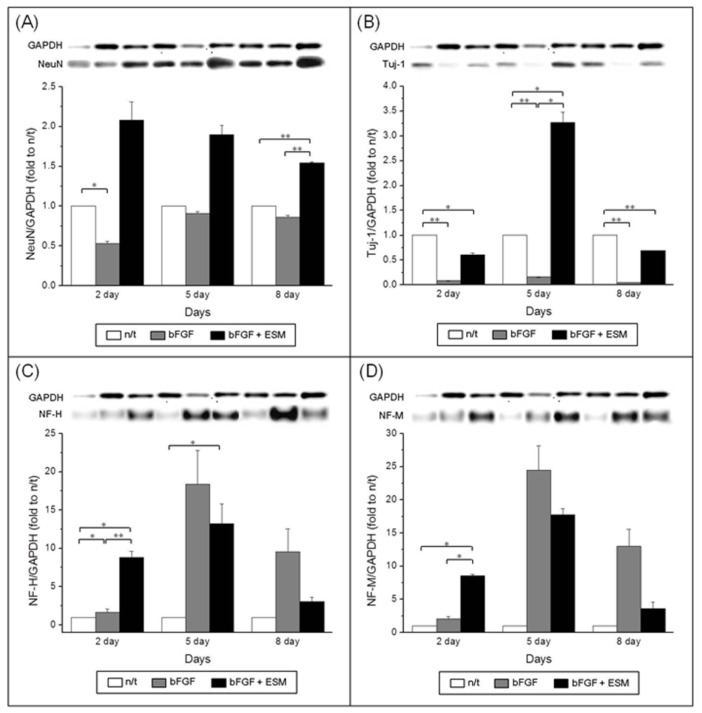
The level of the expressed protein for neuronal markers (**A**, TuJ1; **B**, NeuN) and neurofilaments (**C**, NF-H; **D**, NF-M) was determined by western blot and evaluated by densitometry. Statistical analysis was performed using a one way-ANOVA with Bonferroni’s multiple comparisons (* *p* < 0.05, ** *p* < 0.001).

To confirm the effects of ethosuximide on the neuronal differentiation of rMDSCs, we performed western blotting analyses of the neuron-specific markers NeuN, TuJ1, NF-M, and NF-H. The results of these experiments were consistent with the immunocytochemical analyses of marker expression shown in Figure 3. Western blotting of NeuN, TuJ1, and NF confirmed their expression in the rMDSCs (Figure 4), and the ability of ethosuximide to induce neuronal differentiation was verified by the high levels of NeuN and TuJ1 expression in treated cells (Figure 4A,B). At day 8, the expression of NeuN was significantly higher in bFGF plus ethosuximide-treated cells than in either untreated cells or cells treated with bFGF alone (Figure 4A, *p* < 0.001). Similarly, TuJ1 expression significantly increased by treatment of bFGF-induced rMDSCs with ethosuximide (Figure 4B, *p* < 0.05). These results therefore suggest that ethosuximide treatment induced differentiation of rMDSCs into cells having a neuronal phenotype.

Although the results of this study do not identify the exact mechanism by which ethosuximide induces the neuronal differentiation of rMDSCs, we suggest that ethosuximide may affect cell signaling. As shown in Figures 3H,K and 4C,D, rMDSCs treated with bFGF alone expressed Olig2 and high levels of NF, indicating that bFGF alone induced neural differentiation. However, expression of the neuron-specific proteins NeuN and TuJ1 was only observed in cultures treated with both bFGF and ethosuximide. Thus, we hypothesize that rMDSCs are directed towards the neural lineage by bFGF, and ethosuximide acts synergistically to induce further neuronal differentiation. Ethosuximide has been shown to affect the activity of multiple ion channels, including T-type calcium channels, in vertebrate cultured cells [19,20]. Therefore, it is possible that ethosuximide may induce signaling for neuronal differentiation by binding to such ion channels in rMDSCs.

## 3. Experimental Section

### 3.1. Isolation of Rat MDSCs

All the animals were treated in accordance with the policies and regulations for the care and use of laboratory animals approved by the Institutional Animal Experiment Committee at the College of Medicine, Ajou University. Primary muscle-derived cells were isolated from 4-week-old female Fischer rats by a method described previously [5,6]. In brief, the major hind-limb muscles were removed and enzymatically dissociated with 0.2% collagenase type XI (Sigma, Munich, Germany), dispase (Sigma, Munich, Germany), and trypsin (Gibco BRL, Grand Island, NY, USA). Cells were separated from muscle fiber fragments and tissue debris by differential centrifugation and then resuspended in Dulbecco’s modified Eagle’s medium-high glucose (DMEM; Gibco BRL, Grand Island, NY, USA) containing 10% (*v*/*v*) fetal bovine serum (FBS; Gibco BRL, Grand Island, NY, USA), 100 U/mL penicillin, and 100 μg/mL streptomycin (both Invitrogen, Carlsbad, CA, USA). Cells were seeded into flasks for 1 h, and unattached cells were then transferred into new collagen-coated flasks. The subsequent pre-plates were performed in the next 2 h, 3 h, 1 day, 2 days and 3 days. Finally, the rMDSCs were used at passage 5. The identity of the MDSCs was verified by analyzing CD44 and CD45 expression using a FACScan flow cytometer (Becton Dickinson, Franklin Lakes, NJ, USA) (data not shown).

### 3.2. Neuronal Differentiation of rMDSCs

rMDSCs were cultured in 6-well plates at a density of 5 × 104 cells/well in DMEM containing 5% (*v*/*v*) FBS, 5% (*v*/*v*) horse serum (Gibco BRL, Grand Island, NY, USA), 100 U/mL penicillin, and 100 μg/mL streptomycin. The cells were incubated for 24 h at 37 °C in a 5% CO_2_ atmosphere. For pre-induction of neurogenesis, the cells were cultured for 24 h in amplification medium (DMEM containing 20% (*v*/*v*) FBS supplemented with 25 ng/mL bFGF (Sigma, St. Louis, MO, USA)). The pre-induction medium was then replaced with DMEM alone or DMEM containing 4 mM ethosuximide (Sigma, St. Louis, MO, USA), and neuronal differentiation was monitored for a further 7 days. rMDSCs incubated for 8 days in the medium alone served as negative controls.

### 3.3. Analysis of Cytotoxicity

The effect of ethosuximide on cell proliferation and cell death was measured by performing the 3-(4,5-dimethylthiazol-2-yl)-2,5-diphenyl tetrazolium bromide (MTT; Sigma, St. Louis, MO, USA) assay. The rMDSCs were cultured in 96-well microplates at a density of 5 × 10^3^ cells/well, according to the neuronal differentiation method described above, except that ethosuximide was present at concentration of 1 mM to 8 mM. On days 2, 5, and 8, MTT was added to each well at a final concentration of 1.0 mg/mL. Four hours later, the supernatant was removed and the insoluble formazan crystals were dissolved in 200 μL of dimethylsulfoxide (Sigma, St. Louis, MO, USA). Absorbance was measured at a wavelength of 590 nm. The relative cell proliferation (%) was related to a 100% confluence per well in the A590 test/A590 100% confluence well.

### 3.4. Immunocytochemical Analysis

rMDSCs were cultured in 4-well chamber slides (BD Biocoat, Heidelberg, Germany), according to the neuronal differentiation schedule described above. After 5 days of differentiation, the cells were fixed in 4% paraformaldehyde (Medilab, Seoul, Korea) for 20 min at room temperature. For intracellular staining of neuronal nuclear antigen (NeuN), the cells were permeabilized with 0.1% Triton X-100 in PBS for 15 min at room temperature. The primary antibodies were mouse anti-rat NF-MH antibody (recognizing neurofilament 160 kDa and 200 kDa; Abcam, Cambridge, UK), mouse anti-mouse NeuN antibody (Millipore, Billerica, MA, USA), rabbit anti-rat Olig2 antibody (oligodendrocyte transcription factor 2; Millipore, Billerica, MA, USA), and rabbit anti-rat TuJ1 antibody (neuron-specific class III beta-tubulin; Abcam, Cambridge, UK). The cells were then washed and incubated with secondary Alexa Fluor^®^ 488-conjugated goat anti-rabbit antibody (Invitrogen, Carlsbad, CA, USA) or Alexa Fluor^®^ 594-conjugated donkey anti-mouse IgG antibody (Invitrogen, Carlsbad, CA, USA). Nuclei were counterstained with DAPI nucleic acid stain (4′,6-diamino-2-phenylindol dihydrochloride; Invitrogen, Carlsbad, CA, USA). The cells were examined using a fluorescence microscope AxioObserver Z1 equipped with an ApoTome for optical sectioning (Zeiss, Oberkochen, Germany). To detect specific antibody staining, digital images were captured from each fluorescence channel and superimposed using Axiovision™ software (Zeiss).

### 3.5. Western Blotting Analysis

The cells were incubated for 15 min at 4 °C in RIPA lysis buffer (Cell Signaling Technology, Beverly, MA, USA) and then centrifuged at 12,000 × *g* for 5 min at 4 °C to remove the insoluble material. The supernatants were collected and protein concentrations were determined using a Nanodrop Spectrophotometer (ND-1000; Thermo Scientific, DE, USA). For western blot analysis, lysates (15 μg protein per sample) were resolved by electrophoresis in NuPAGE^®^ Novex^®^ 4%–12% Bis-Tris gels (Invitrogen, Carlsbad, CA, USA), and proteins were transferred to nitrocellulose membranes (iBlot^®^ Transfer Stack, Invitrogen, Carlsbad, CA, USA). The membranes were probed using antibodies to NF-M, NF-H (both Abcam, UK), NeuN (Millipore, Billerica, MA, USA), TuJ1 (Abcam, Cambridge, UK), and rat glyceraldehyde 3-phosphate dehydrogenase (GAPDH; Santa Cruz Biotechnology, CA, USA) Horseradish peroxidase-conjugated sheep anti-rabbit antibody (Santa Cruz Biotechnology, Santa Cruz, CA, USA) or horseradish peroxidase-conjugated goat anti-mouse IgG (Novus Biologicals, Littleton, CO, USA) were used as secondary antibodies. The reaction products were visualized by chemiluminescence using an ECL western blotting detection kit (GE Healthcare, Milwaukee, WI, USA). Quantification of band densities was performed with an ImageQuant LAS 4000 mini luminescent image analyzer (GE Healthcare, Milwaukee, WI, USA).

### 3.6. Statistical Analysis

The results of the cytotoxicity assays and western blotting analyses are given as the mean and standard deviation (SD) of 3 independent experiments. The results were analyzed for statistical differences by one-way ANOVA with Bonferroni’s correction for multiple comparisons, using the Prism 3.0 software package (GraphPad Software Inc., San Diego, CA, USA). A p value of 0.05 was considered to be statistically significant.

## 4. Conclusions

Our data demonstrate that rMDSCs differentiated primarily into neural-like cells in the presence of bFGF and ethosuximide. In particular, the bFGF-induced expression of neuron-specific proteins was amplified in the presence of ethosuximide. These results support the idea that human MDSCs could also be induced by ethosuximide to undergo neuronal differentiation. Although, to the best of our knowledge, we provide the first evidence that treatment with bFGF and ethosuximide induces neural differentiation of MDSCs *in vitro*, further studies are needed to exploit differentiation in the longer time, to investigate functional evidence of differentiated cells and to determine the molecular mechanisms underlying the differentiation process. In addition, we need further studies using PCR analyses to confirm the oligodendrocytes gene in immunohistochemistry.
